# Selective diagonal-free ^13^C,^13^C-edited aliphatic–aromatic NOESY experiment with non-uniform sampling

**DOI:** 10.1007/s10858-013-9739-5

**Published:** 2013-05-09

**Authors:** Jan Stanek, Michał Nowakowski, Saurabh Saxena, Katarzyna Ruszczyńska-Bartnik, Andrzej Ejchart, Wiktor Koźmiński

**Affiliations:** 1 Faculty of Chemistry, University of Warsaw, Pasteura 1, 02093 Warsaw, Poland; 2 Institute of Biochemistry and Biophysics, Polish Academy of Science, 02106 Warsaw, Poland

**Keywords:** Aliphatic–aromatic NOESY, Proteins, Selective pulses, Four-dimensional NMR, Non-uniform sampling

## Abstract

A band-selective aromatic–aliphatic C,C-edited four-dimensional NOESY experiment is proposed here. Its key advantage is the absence of auto-correlation signals which makes it very attractive for joint use with non-uniform sampling. It is demonstrated here that the sensitivity of the experiment is not significantly affected by utilization of selective pulses (for either aromatic-13C or aliphatic-13C spins). The method was applied to the sample of E32Q mutant of human S100A1 protein, a homodimer of total molecular mass ~20 kDa. High-resolution 4D spectra were obtained from ~1.5 % of sampling points required conventionally. It is shown that superior resolution facilitates unambiguous assignment of observed aliphatic–aromatic cross-peaks. Additionally, the addition of aliphatic-13C dimension enables to resolve peaks with degenerated aliphatic ^1^H chemical shifts. All observed cross-peaks were validated against previously determined 3D structure of E32Q mutant of S100A1 protein (PDB 2LHL). The increased reliability of structural constraints obtained from the proposed high-resolution 4D 13C(ali),13C(aro)-edited NOESY can be exploited in the automated protocols of structure determination of proteins.

**Electronic supplementary material:**

The online version of this article (doi:10.1007/s10858-013-9739-5) contains supplementary material, which is available to authorized users.

## Introduction

Modern multidimensional NMR spectroscopy allows successful determination of three-dimensional structure for double- or triple-labeled proteins with molecular weights of up to 50 kDa (Kay 2005). Typically protein structure elucidation can be split into two main parts (Wüthrich 1986): sequence-specific assignment of chemical shifts, and extraction of distances between proton pairs from NOESY spectra. The precision of determined structures depends primarily on a number of observed NOE contacts and their proper assignment. The main difficulty arises due to the ambiguity of NOE assignment since chemical shifts are derived from NMR measurements with a certain level of precision. A related severe problem lies in the frequent chemical shift degeneracy in proteins. It was shown that in larger systems less than 10 % of NOE cross peaks can be assigned unambiguously (Mumenthaler et al. 1997). Over the years several dedicated protocols for automatic NOE assignment were developed (Gronwald et al. 2002; Herrmann et al. 2002b; Nilges and O’Donoghue 1998). Nevertheless, the problem of structural calculations from ambiguous NOEs still remains severe. Here we report an NMR measurement that allows to dramatically decrease the ambiguity of assignment of NOE between aromatic and aliphatic protons. It was shown previously (Jee and Guntert 2003) that chemical shifts of aromatic side chains are crucial for NOE assignment. These residues are buried in the hydrophobic core of a protein and give rise to many long-range NOE contacts. The same conclusions concern protons of methyl groups (Janin et al. 1988). Therefore, the ability to obtain unambiguous contacts among aromatic and aliphatic protons can be a valuable tool for determination of protein structure with improved precision. The intrinsic uncertainty of NOE assignment due to flips of aromatic rings and frequently degenerated chemical shifts is alleviated by introduction of pseudo-atoms or other solutions (Wüthrich et al. 1983); the unavoidable loss of structure accuracy is entailed.

Although three-dimensional NOESY methods are most commonly applied (Zhang et al. 1994; Muhandiram et al. 1993), the full potential of ^15^N,^13^C isotope labeling can be utilized with double-heteronuclear-edited 4D spectra (Luan et al. 2005). It should be emphasized that the reasonable resolution of these spectra can be preserved by employing non-uniform sampling (NUS) of indirect time domains. Due to the strict requirements regarding high dynamic range and linearity of peak intensities, NUS NOESY spectra are particularly demanding for processing methods; this is reflected by a limited number of applications published to date (Luan et al. 2005; Hiller et al. 2009; Coggins et al. 2012; Stanek and Koźmiński 2010).

Diagonal-free NOESY experiments have attracted a considerable attention since the spectral reconstruction from sparse data is less severe for spectra with a limited dynamic range of signal amplitudes. Recently, a non-uniformly sampled (NUS) 4D ^15^N,^15^N-edited TROSY-NOESY-TROSY experiment based on the orthogonal spin state selection was reported (Werner-Allen et al. 2010; Diercks et al. 2010). The utility of an alternative approach to the diagonal peak suppression based on the straightforward diagonal subtraction (Wu et al. 2004) was also demonstrated for methyl–methyl NOESY spectroscopy (Wen et al. 2012). In these methods, however, the sensitivity per unit of time is reduced by the non-negligible factor of 2.

The ^13^C,^15^N-edited 4D NOESY experiment (Stanek et al. 2012) is an example of the most straightforward approach to the acquisition of diagonal-free NOESY spectra. Of the particular relevance is that it does not imply any loss of sensitivity.

In this work, we propose a novel four-dimensional aliphatic–aromatic NOESY experiment in which diagonal peaks are efficiently suppressed by the selective manipulation of ^13^C spins of different kinds. The major aim of this experiment is to retain high sensitivity of the method while achieving a reasonable level of selectivity and associated diagonal peak suppression. The proposed method complements the set of recently reported diagonal-free 4D aliphatic–amide (Stanek et al. 2012), amide–amide (Werner-Allen et al. 2010) and aliphatic–aliphatic (Wen et al. 2012) NUS NOESY experiments.

## Materials and methods

### Selective 4D HMQC-NOESY-HSQC experiments

**Fig. 1:**
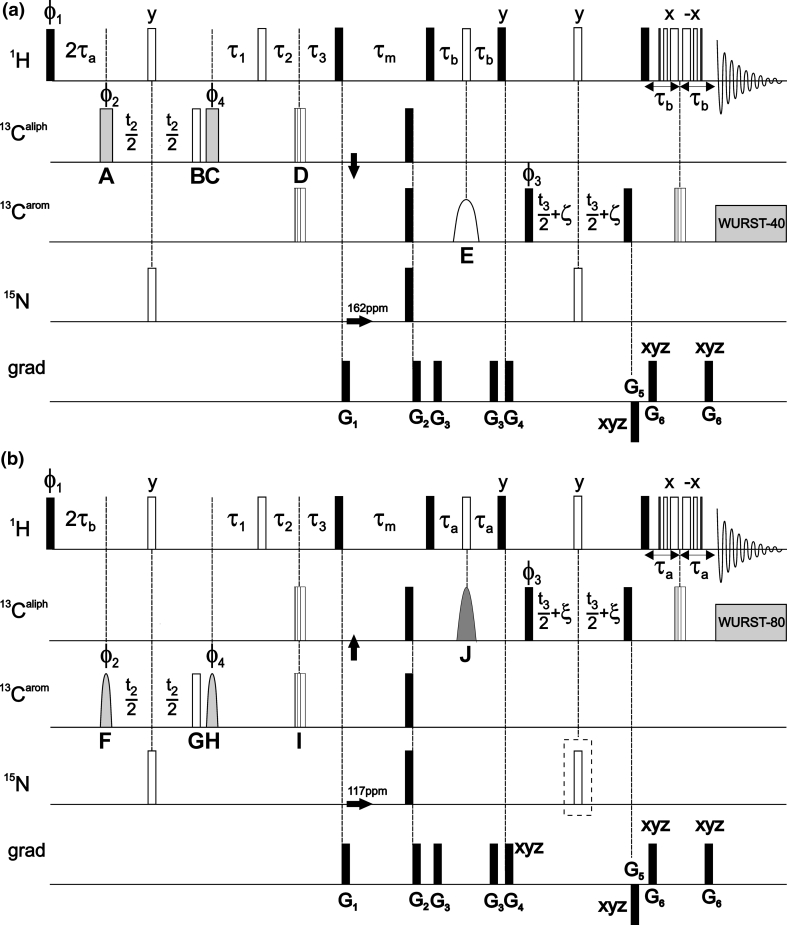
Pulse sequence scheme for (**a**) C(aliph)HMQC-NOESY-C(aro)HSQC and (**b**) C(aro)HMQC-NOESY-C(aliph)HSQC. 90° and 180° ‘hard’ pulses are represented by *filled* and *open bars*, respectively. *Shaped pulses* are represented as follows: (**a**) 40 ppm selective pulse of the iburp-2 profile (Geen and Freeman 1991) (659 μs duration, 7.6 kHz peak r.f. field) is shown as *wide open bell-shaped pulse* (denoted ‘*E*’). *Wide grey rectangular pulses* (‘*A*’ and ‘*C*’) are of duration of 61 μs and peak r.f. 4.1 kHz (calibrated to give null excitation 90 ppm off -resonance). (**b**) *Wide grey bell-shaped pulse* denotes 80 ppm-selective hyperbolic secant adiabatic pulse (Silver et al. 1984) of duration of 1 ms (r.f. peak 6.9 kHz). *Narrow grey bell-shaped pulses* (‘*F*’ and ‘*H*’) utilize Gaussian profile (Bauer et al. 1984) (truncated at 1 %, duration of 178 μs, peak r.f. 3.4 kHz, ca. 60 ppm bandwidth). Note that for some hardware the shaped 90° pulses (‘*A*’, ‘*C*’, ‘*F*’ and ‘*H*’) may require small angle phase adjustment to compensate 0th order phase shift with respect to refocusing pulses (‘*B*’ and ‘*G*’) which are applied at full power. ^15^N refocusing pulse enclosed in *dashed-line box* (sequence **b**) is optional and may be used if delay complementing to half-dwell time, ξ = [(sw3)^-1^ − pw180(N)]/2, is positive. Otherwise ξ = [(sw3)^-1^ − pw180(H)]/2. Similar rules apply to the delay ζ for sequence (**a**). Refocusing of C_aliph_-C’ couplings in *t*_2_ (**a**) or *t*_3_ (**b**) may be considered if max. evolution time of aliphatic ^13^C spins exceeds 9 ms. The composite 34.2°_−x_123°_x_197.6°_−x_288.8°_x_ pulse (‘*D*’ and ‘*I*’) was used in HMQC for broadband inversion of ^13^C spins (Shaka 1985). WATERGATE (Piotto et al. 1992) with 3-9-19 pulses separated by (3.2 kHz)^−1^ was employed for experiment (**a**). ^13^C composite pulse decoupling was performed employing WURST scheme (Kupče and Freeman 1995). The durations of ‘hard’ π/2 pulses were 7.1, 14.3 and 31 μs for ^1^H, ^13^C and ^15^N, respectively. ϕ_1_, ϕ_2_ and ϕ_3_ are incremented for quadrature detection in *t*_1_, *t*_2_ and *t*_3_ using States (ω_1_) or States-TPPI (ω_2_, ω_3_) method. Four-step phase cycle is as follows: ϕ_1_ = 45°; ϕ_2_ = *x*, −*x*; ϕ_3_ = 2(*x*), 2(−*x*); ϕ_4_ = 2(*x*), 2(−*x*); ϕ_5_ = 135°; ϕ_rec_ = *x*, −*x*, *x*, −*x*. Delays are set as follows: τ_a_ = 1.79 ms ≈ (4 J_CHaliph_)^−1^, τ_b_ = 1.56 ms ≈ (4 J_CHarom_)^−1^. NOESY mixing time τ_m_ = 150 ms was used. For the semi-constant time evolution in *t*_1_ (^1^H) the delays τ_1_, τ_2_ and τ_3_ are *t*_1_/2, *t*_1_(1 − 2Δ/*t*_1,max_)/2 and Δ(1 − *t*_1_/*t*_1,max_), where Δ = 2τ_a_, or Δ = 2τ_b_ for sequences (**a**) and (**b**), respectively (Stanek et al. 2012). Gradient levels and durations are: G_1_ (2 ms, 6.5 Gs/cm), G_2_ (2 ms, 14.2 Gs/cm), G_3_ (0.5 ms, 1.77 Gs/cm), G_4_ (2 ms, 11.3 Gs/cm), G_5_ (2 ms, −12.9 Gs/cm), G_6 _(0.5 ms, 5.4 Gs/cm). Proton carrier frequency was set on resonance with water (4.68 ppm), carbon carrier was set to 35 ppm and switched to 125 ppm as indicated by the vertical arrow; ^15^N carrier was set to 117 ppm and shifted to 162 ppm at the beginning of NOESY mixing period. For the aliphatic-to-aromatic NOESY (**a**) 4,400 sampling points (t_1_,t_2_,t_3_) were randomly chosen from 120 × 84 × 30 Cartesian grid according to Gaussian probability distribution, *p*(*t*) = exp[−(*t/t*_max_)^2^/2σ^2^], σ = 0.5, with Poisson disk restrictions (Kazimierczuk et al. 2008). Maximum evolution times of 15 (*t*_1_), 6 (*t*_2_) and 5 ms (*t*_3_) were achieved in the indirectly detected dimensions. Spectral widths of 8, 14, 6 and 12 kHz were set in ω_1_, ω_2_, ω_3_ and ω_4_ dimensions, respectively. In the full 4D spectrum any *residual* diagonal peaks can be folded in ^13^C (ω_2_ and ω_3_) dimensions without the risk of overlap and misinterpretation with genuine peaks. The only requirement is to ensure proper ^1^H (ω_1_) spectral width to avoid aliasing in this indirect dimension. The restriction of ^13^C spectral widths is very practical as it saves vast amounts of disk space. Inter-scan delay of 1.2 ms was used. The total experimental time was 57 h

The pulse sequences of 4D experiments providing aromatic–aliphatic NOESY cross-peaks are shown in Fig. 1. Two possible solutions are discussed here: aliphatic-to-aromatic (Fig. 1a) and aromatic-to-aliphatic (Fig. 1b) HMQC-NOESY-HSQC. In both cases the optimized HMQC scheme (Stanek et al. 2012) was incorporated for *t*_1_ (^1^H) and *t*_2_ (^13^C) evolution as it utilizes the multiple-quantum line narrowing effect for C–H coherences (Grzesiek et al. 1995). In the previous work on 3D H(aro)-NOESY-CH_3_,H_N_ and 3D C(aro)-NOESY-CH_3_,H_N_ (Xia et al. 2001) the refocused INEPT was utilized for the selection of protons coupled to aromatic ^13^C spins. In this case selective transfer can be achieved employing shaped *inversion* pulses and pulsed field gradients (PFGs) that suppress undesired coherences in the subsequent *z*-filtration. We have found that the same task is far more demanding using HMQC. In this case PFGs cannot be used to distinguish aromatic and aliphatic MQ ^1^H–^13^C coherences as both have equal coherence order after initial 1/2J_CH_ delay and ‘hard’ excitation pulse. If the selective excitation pulse is employed PFGs can be useful, however, sensitivity loss of a factor of $\sqrt 2$ is incurred and additional time is required for gradient encoding when ^13^C magnetization is transverse. We thus conclude that the optimal solution includes phase cycling of selective excitation pulses. Despite these difficulties HMQC is preferred over refocused INEPT owing to (1) the apparently slower relaxation of MQ coherences and (2) the possibility to incorporate both magnetization transfer delays (1/J_CH_ in total) for the shared-time proton evolution (*t*_1_) that leads to increased sensitivity (Stanek et al. 2012).

Here, the selection of either aliphatic (Fig. 1a) or aromatic (Fig. 1b) proton-carbon MQ coherences is accomplished *solely* by band-selective excitation of ^13^C spins in conjunction with the four-step phase cycle of two π/2 pulses (‘A’, ‘C’, or ‘F’, ‘H’, respectively). Therefore, the choice of selective π/2 pulses is of the particular relevance. The simulations of excitation profiles performed using Spinach software (Hogben et al. 2011) were fairly beneficial for this purpose (see Supplementary Materials). Apparently, it is quite straightforward *not* to excite aromatic ^13^C spins (the sequence shown in Fig. 1a) and simple rectangular pulses of properly adjusted γB_1_ can be employed. More advanced pulses such as Gaussian cascade Q5 (Emsley and Bodenhausen 1992) or e-BURP (Geen and Freeman 1991) could be utilized as well, however, these pulses are (1) of significantly larger duration, (2) more sensitive to miscalibration and (3) require high peak r.f. field. It was experimentally verified that these shortcomings offset theoretical benefits of high quality pulses and significant loss of sensitivity is incurred. For the aromatic-HMQC (Fig. 1b) short Gaussian π/2 pulses proved to be optimal.

Due to the use of shaped π/2 ^13^C pulses in HMQC the refocusing π pulse is obligatory for correct phase properties of signals in ω_2_ dimension. However, no additional selectivity improvement can be achieved by replacement of this pulse by a band-selective one. Similarly, the subsequent carbon inversion pulse in HMQC (denoted ‘D’ and ‘I’, respectively) should be sufficiently broadband. A shaped pulse here would only result in the *t*_1_-dependent *J*_*CH*_ evolution of unaffected coherences during total time of τ_1_ + τ_2_ − τ_3_ = *t*_1_ + 2τ_a_. The resulting signals would be impossible to suppress using neither phase cycling nor pulsed field gradients.

### Experimental

All NMR experiments were performed for the E32Q mutant of human S100A1 protein, for which resonance assignments are available in BMRB under accession code 17857. The high resolution 3D structure based on the standard 3D ^13^C- and ^15^N-edited NOESY-HSQC spectra (Zhang et al. 1994; Muhandiram et al. 1993) is available in PDB under accession code 2LHL. Similarly to the native S100A1 (Nowakowski et al. 2011), this protein mutant also exists in solution as a homodimer built up of noncovalently attached subunits (molecular weight of the monomer is 10 kDa). NMR sample contained 1.0 mM ^15^N,^13^C labeled protein (the monomer concentration) in 90 %/10 % H_2_O/D_2_O, 50 mM TRIS-d_11_, 1.0 mM EDTA, 0.1 mM NaN_3_ and 50 mM NaCl with pH adjusted to 7.2.

The experiments were performed at 37 °C on a 700 MHz Agilent/Varian spectrometer equipped with the Performa XYZ PFG unit and the 5 mm ^1^H,^13^C,^15^N triple-resonance probehead.

3D NOESY-HSQC and 4D HMQC-NOESY-HSQC spectra were analyzed with SPARKY (Goddard and Kneller). In both cases, peak picking and inspection of resulting peak lists was performed in approximately 30 min. Conventionally sampled 3D NOESY-^13^C-HSQC spectrum was acquired in 36 h and processed using nmrPipe program (Delaglio et al. 1995).

To derive NOESY interproton distance constrains from the 4D ^13^C(ali),^13^C(aro)-edited NOESY, automatic NOESY assignment procedure (Herrmann et al. 2002a) implemented in program CYANA 3.0 (Guntert et al. 1997) was applied. The final validation of obtained constraints was performed with XPLOR-NIH program (Schwieters et al. 2003).

The processing of four-dimensional NUS data was accomplished by the home-written SSA (signal separation algorithm) software package, which can be downloaded free of charge for non-commercial purposes from the website http://nmr.cent3.uw.edu.pl (64-bit versions for Linux). Artefact suppression in SSA software (*cleaner4d* program) was intentionally disabled by setting maximum number of iterations to zero. This is equivalent to the use of raw zero-augmented Fourier Transformation and limits *cleaner4d* to data conversion. The actual computation of spectral data was performed using *reconstructor4d* program (also from SSA package) that implements (a) automatic choice between FFT and DFT whenever beneficial, (b) multi-threaded computations and (c) memory-mapped and asynchronous input–output disk operations to improve overall performance. The total processing time on a PC equipped with Intel i5 3.3 GHz CPU running under Ubuntu 11 was 2 min and 6 min 30 s using 4 and 1 threads, respectively. Final spectrum stored in Sparky format (single-precision) was of size of 10.1 GB. SSA software supports native Agilent/Varian as well as nmrPipe format as input data. Further information on using SSA software can be found in the Supplementary Material.

## Results and discussion

**Fig. 2:**
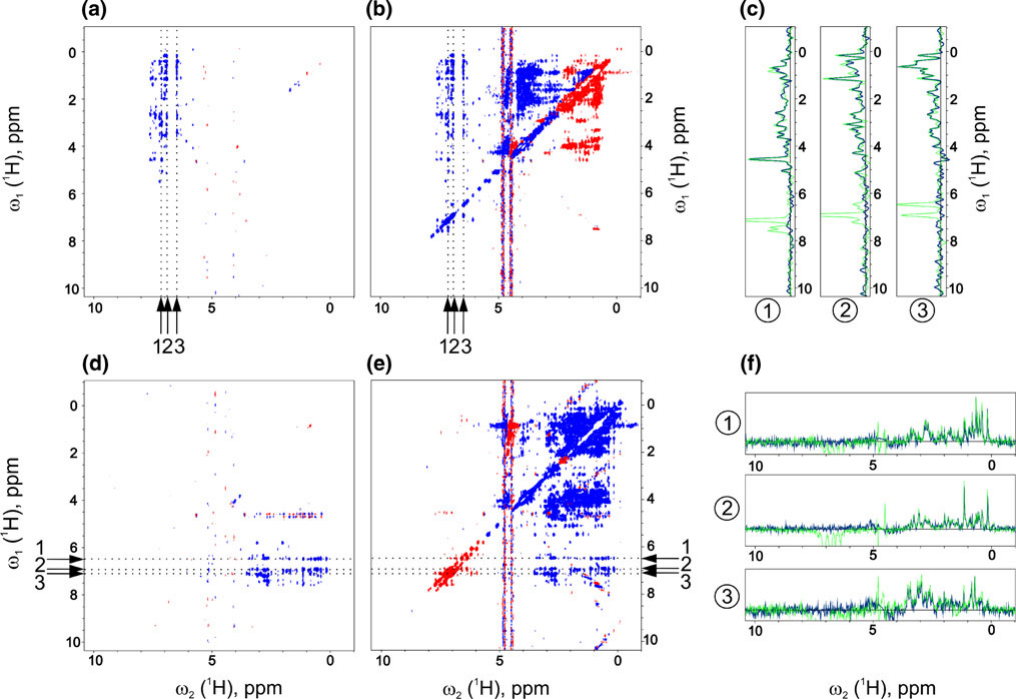
The comparison of homonuclear (ω_1_ (^1^H)–ω_2_ (^1^H)) versions of (**a**) selective aliphatic-to-aromatic, (**b**) non-selective aliphatic-to-aromatic (see Fig. 1b), (**d**) selective aromatic-to-aliphatic and (**e**) non-selective aromatic-to-aliphatic NOESY experiments. Spectra (**a**) and (**d**) were acquired using pulse sequences shown in Fig. 1a, b, respectively. Non-selective versions (**b**) and (**e**) utilize the same pulse sequences as (**a**) and (**d**), respectively, with all the *shaped pulses* replaced by ‘hard’ ones. ^13^C spins were not evolved. Spectra (**b**) and (**e**) show that non-selective experiments suffer from many spectral artefacts due to poor broadband performance of ‘hard’ pulses and decoupling schemes at high fields. Apart from sign inversions across the spectrum, intense axial peaks as well as phase-distorted pseudo-diagonals are present (**e**). Selective experiments (**a**, **d**) achieve to filter-out virtually all diagonal signals with only slight decrease of intensity of cross-peaks. (**c**) Superimposed 1D cross-sections across ω_1_ (^1^H) from spectra (**a**, *blue curves*) and (**b**, *green curves*) for ω_2_ coordinates indicated by the *vertical arrows* (*1*–*3*). (**f**) ω_2_ (^1^H, direct dimension) cross-sections of spectra (**d**, *blue*) and (**e**, *green curves*) at ω_1_ coordinates indicated by the *horizontal arrows* (*1*–*3*). The cross-sections are plotted using the same intensity scale, thus they enable direct comparison of experimental sensitivity between selective and non-selective versions of experiments (**a** vs. **b**, and **d** vs. **e**). For each experiment 120 increments with 40 scans were collected (duration of 3 h). Spectral width of 8 kHz in the indirect ω_1_ (^1^H) dimension was set

The performance of proposed selective NOESY experiments was first verified on conventionally sampled homonuclear 2D spectra (Fig. 2). For both possibilities considered here, namely aliphatic-to-aromatic (Fig. 2a) and aromatic-to-aliphatic NOESY (Fig. 2d) excellent suppression of undesired autocorrelation (i. e. aliphatic–aliphatic and aromatic–aromatic) signals was obtained. Noteworthy is that in the non-selective versions of these experiments (i.e. without use of shaped pulses) substantial amount of spectral artefacts appear that may distort relevant region of aromatic–aliphatic cross-peaks (see, e.g., pseudo-diagonals in Fig. 2e). Another conclusion is that due to the poor broadband performance of ^13^C r.f. pulses and decoupling schemes at high static magnetic fields the *simultaneous* acquisition of aromatic–aliphatic and aliphatic–aromatic NOESY cross-peaks is hardly achievable (Fig. 2b, e). Thus, selection of one possibility is not lossy in terms of structural information gained.

The sensitivity of NOESY experiments is a critical issue. 2D spectra shown in Fig. 2 do not indicate any substantial signal degradation resulting from use of selective pulses. Indeed, the closer inspection of 1D traces showing NOESY cross-peaks (Fig. 2c, f) reveals only minor decrease of sensitivity accompanied by virtually complete suppression of diagonal peaks. This conclusion applies to both 2D H–H correlation spectra: ^13^C(aro)-^13^C(ali)- and ^13^C(ali)-^13^C(aro)-filtered. A rough quantification was performed using integration over regions of aliphatic–aromatic cross-peaks. Although quite imprecise and susceptible to presence of unrelated spectral artefacts (as in Fig. 2e), the integration suggests that selective spectra (Fig. 2a, d) are approximately up to 10 % less intense than their non-selective counterparts (Fig. 2b, e). In the context of non-uniform sampling employed for four-dimensional experiment a rather moderate price for elimination of strong diagonal peaks—and associated spectral artifacts—seems fairly acceptable.

While both experimental possibilities presented here (Fig. 1a, b) seem to provide equivalent structural information, there is a number of practical considerations involved. Since aliphatic protons are typically far more populated than aromatic ones high resolution in aliphatic ^1^H dimension is especially desired. This suggests to prefer the aromatic-to-aliphatic variant which provides the optimal resolution of directly detected ^1^H^aliph^ dimension. In the alternative aliphatic-to-aromatic spectrum the indirectly detected ^1^H^aliph^ dimension features lower resolution, however, the assignment is supported by additional ^13^C^aliph^ chemical shift. Noteworthy, aliphatic ^1^H and ^13^C nuclei exhibit a broader distribution of chemical shifts than aromatic ones, thus simplifying the assignment task. To sum up, there seems to be no evident resolution advantage of one variant over another.

Frequently, samples in H_2_O/D_2_O solution are used also for acquisition of NOESY spectra in order to avoid data inconsistency due to different sample preparation and isotopic effects. In this case the suppression of solvent signal becomes a major concern. The evident advantage of ^13^C^ali^,^13^C^aro^-separated NOESY is that WATERGATE (Piotto et al. 1992) can be employed without suppression of relevant resonances. On the other hand, for the ^13^C^aro^,^13^C^ali^-separated NOESY presaturation of HDO signal can be employed, however, our experience shows limited benefits of this approach.

It is also worth mentioning that in the case of the ^13^C^ali^,^13^C^aro^-NOESY the detection of aromatic protons decreases requirements on ^13^C decoupling during acquisition. As broadband decoupling at high *B*_0_ field can be quite detrimental for the probehead circuits, the ^13^C^ali^,^13^C^aro^-edited NOESY might be easier to implement on some hardware (e.g., cryoprobes).

The most decisive argument in favor of the ^13^C^ali^,^13^C^aro^-separated NOESY is its higher sensitivity. Although it is not manifested in 2D H–H versions of compared experiments (Fig. 2a, d show similar intensity of cross-peaks), the direct comparison of four-dimensional C,C-edited spectra indicated that ^13^C^ali^,^13^C^aro^-edited experiment is considerably more sensitive. As the effect of shaped pulses is similar for both sequences, the most likely reason for different sensitivity of these experiments is the different attenuation of transverse relaxation rate observed for multiple quantum aromatic and aliphatic C–H coherences stored during ^13^C *t*_2_ evolution period.

**Fig. 3:**
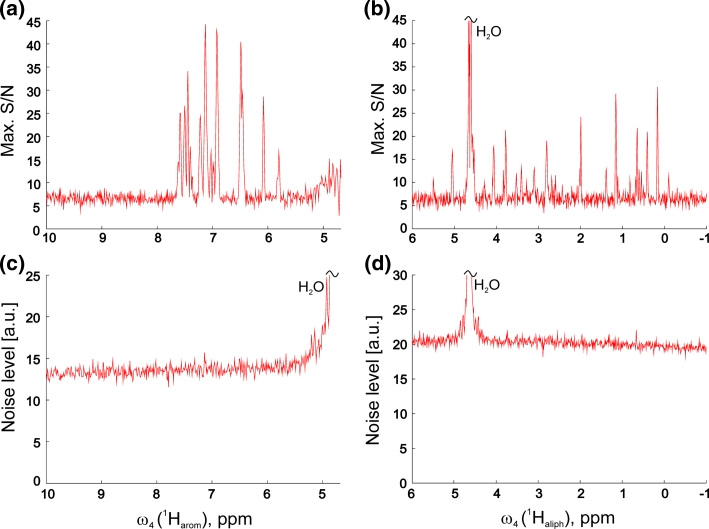
**a**, **b** Maximum signal-to-noise ratio measured for peaks in the individual 3D cubes (indirect domains ω_1_ × ω_2_ × ω_3_) in each point of directly detected dimension (ω_4_). The *plots* show the ratio of the most intense spectral point (absolute value) to the noise level in the corresponding 3D cube (**c**, **d**). Assuming that cross-peaks do not overlap in the 4D spectrum these *plots* reflect the overall sensitivity of experiment. Considerable sensitivity advantage of the aliphatic-to-aromatic (**a**, **c**) over aromatic-to-aliphatic (**b**, **d**) NOESY is apparent. Noise levels (**c**, **d**) in the 3D cubes for each point of directly detected dimension were measured as the median of absolute values of all spectral intensities. From the uniformity of noise level across directly detected dimension one can conclude that advanced procedures for NUS artifact suppression are not beneficial in this case, and that the both experiments are sensitivity-limited. The scale was cut for intense water resonance (at 4.68 ppm)

The distinguishing feature of selective aliphatic–aromatic NOESY is the absence of strong diagonal signals which provide no additional structural information. This has a crucial implication that resulting spectra acquired using non-uniform sampling are not distorted by sampling artefacts, or their impact can be neglected in comparison to thermal noise (see the flat baselines of noise in Fig. 3c, d). Therefore, more advanced processing schemes such as SSA or CLEAN do not have a particular advantage over zero-augmented Fourier Transformation (Kazimierczuk et al. 2012). The practical consequence is that a full 4D spectral matrix can be rapidly reconstructed, usually in a few minutes, depending mostly on hard disk performance (see “Experimental” section). Additionally, the S/N of most intense cross-peaks found in the indirect 3D cubes ω_1_–ω_3_ shown in Fig. 3a, b confirms the above mentioned difference in overall sensitivity of two variants of selective NOESY.

**Fig. 4:**
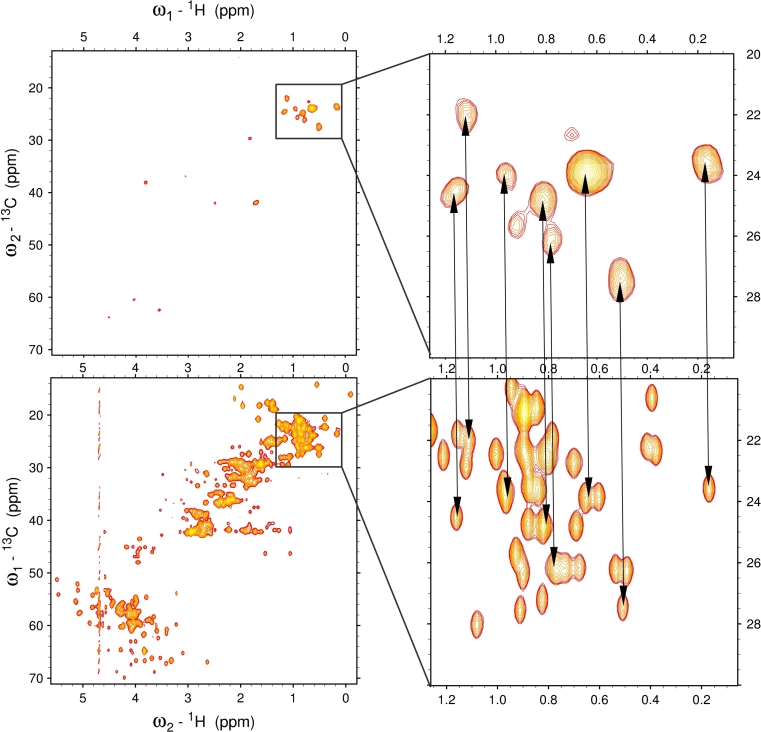
Schematic illustration of reliable cross-peak assignment strategy using 4D C(ali),C(aro)-edited NOESY spectrum. In the *top left panel* a plane of 4D ^13^C(ali),^13^C(aro)-edited NOESY at Tyr 74 Hε/Cε chemical shifts is presented with expanded methyl region of the spectrum on the *right side*. In the *bottom left panel* 2D ^1^H–^13^C HSQC spectrum is shown with expanded methyl region on the *right side*. Corresponding cross peaks in the cross-section of 4D spectrum and the methyl region of HSQC are connected by *arrows*. Relevant methyl-aromatic cross-peaks can be readily assigned (even manually) as the ambiguity is removed by additional aliphatic-^13^C chemical shift (ω_2_)

E32Q mutant of human S100A1 protein was used to demonstrate the advantage of 4D ^13^C(ali),^13^C(aro)-edited NOESY in identification of NOEs between aromatic and aliphatic protons. 4D NOESY spectrum can be conveniently analyzed with commonly used SPARKY software. After synchronization of the ω_3_ and ω_4_ axes with the aromatic region of ^13^C–HSQC, the peak picking can be performed manually. An example in Fig. 4 shows a set of NOEs between Tyr 74 Hε and methyl protons. Majority of these correlations can be unambiguously assigned by visual comparison of particular aliphatic planes of 4D NOESY and the aliphatic region of ^13^C-HSQC spectrum.

**Fig. 5:**
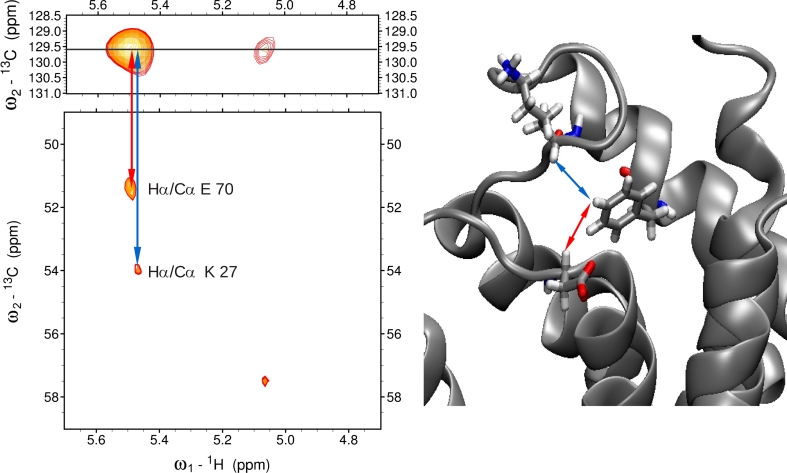
An example showing removal of proton chemical shift degeneracy by addition of aliphatic carbon-13 dimension. In the standard 3D ^13^C^aro^-edited NOESY-HSQC spectrum (*top left panel*) only two cross peaks are observed for *Hζ* proton of Phe 15. In the *bottom left panel* a cross section from 4D aliphatic–aromatic NOESY is presented, and one of the cross peaks splits into two. On the *right panel* the corresponding fragment of previously determined 3D structure of S100A1 E32Q (PDB code 2LHL) is shown. All three atoms giving rise to NOE contacts are close in space. For clarity only the E70 Hα–F15 Hζ and K27 Hα–F15 Hζ interactions were marked

It should be emphasized that most of proton chemical shift degeneracies observed in 3D ^13^C(aro)-edited NOESY has been removed in 4D ^13^C(ali),^13^C(aro)-edited NOESY providing 21 new reliable structural constraints. The example of elimination of degeneracy is presented in Fig. 5. The cross peak present in the 3D NOESY spectrum splits into two NOE correlations in the 4D spectrum. Both observed correlations are fully consistent with the 3D protein structure (cf. the right panel of Fig. 5).

**Fig. 6:**
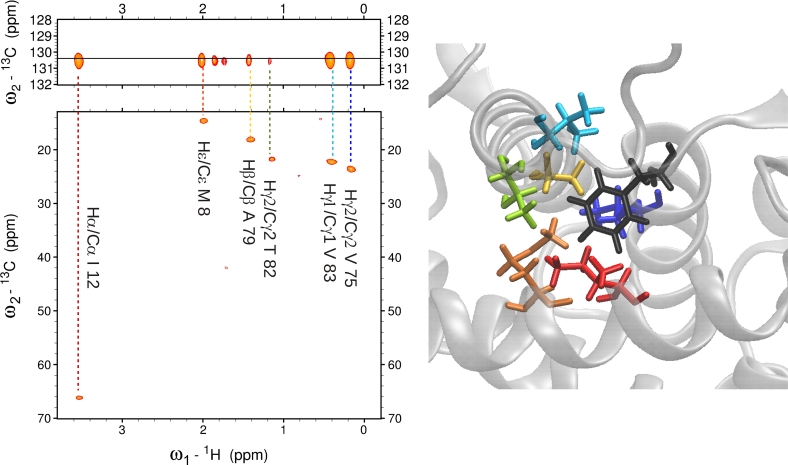
In the *top left panel* the slice of 3D ^13^C-edited NOESY-HSQC for Hε/Cε of Phe 15 is presented. In the *bottom left panel* corresponding plane of 4D ^13^C(ali),^13^C(aro)-edited NOESY is presented. In the *right panel* a fragment of 3D structure derived previously is presented. All atoms giving rise to NOEs are in proximity. Residues are *color-coded* as are the *lines* connecting corresponding peaks in the spectra, while Phe 15 is presented in *black*

All constraints obtained from the 4D NOESY spectrum were carefully validated against the previously determined structure of the E32Q S100A1 protein (PDB accession code 2LHL). As demonstrated for Hε/Cε of Phe 15, all nuclei contributing to observed NOEs are in spatial proximity (cf. the right panel of Fig. 6). Additionally, it was verified that most cross-peaks identified in the 4D spectrum were assigned consistently with peaks present in its 3D counterpart. The only few differences concern stereospecific assignment of NOE contacts and peaks that have not be assigned in 3D spectrum due to ambiguity.

At present manual NOESY cross peak assignment is rarely performed. Therefore, the standard automatic protocol for assignment of NOE correlations, implemented in CYANA software, was applied for the analysis of the 3D ^13^C(aro)-edited NOESY-HSQC and 4D ^13^C(ali),^13^C(aro)-edited NOESY spectra. The results are given in Table 1. It seems surprising that 3D spectrum resulted in a slightly larger number of constraints than the 4D one. It should be borne in mind, however, that most of 3D-derived NOE correlations in larger proteins are heavily degenerated. Current assignment algorithms deduce such a distance constraint on the basis of chemical shifts and the supposed proton spatial proximity in the tentative structure model. Consequently, a number of correlations are, to some extent, speculative and estimation of their intensities (necessary for distance calibration) is putative. On the other hand, most of 4D-derived NOE correlations are uniquely and unambiguously identified and can be properly quantified. It might be of crucial importance for de novo structure determination of an unknown system. As shown in Table 1 some weak correlations visible in 3D NOESY were missing in the 4D spectrum due to its intrinsically lower sensitivity, however, these include mostly weak intraresidual H^α^–H^δ^, H^α^–H^ε^ and H^β^–H^ε^ NOE contacts. Most importantly, the number of essential long range and intersubunit constraints was not affected.

**Table 1:** The summary of NOE constraints derived from standard 3D ^13^C-edited NOESY HSQC (tuned to aromatic carbons) and 4D ^13^C(ali),^13^C(aro)-edited NOESY

	3D ^13^C(aro)-edited NOESY HSQC	4D ^13^C(ali),^13^C(aro)-edited NOESY	Number of common constraints from 3D and 4D spectra
Constraints between aromatic and aliphatic protons	156	131	111
Intraresidual (i − j ≤ 1)	63	50	44
Medium range (1 < i − j < 5)	22	14	10
Long range (i − j ≥ 5)	45	44	37
Intersubunit	26	23	20

4D spectrum provided 20 additional constraints that could not be assigned in the 3D spectrum. A number of weak NOE contacts present in 3D spectrum were not detected in 4D NOESY, including mostly intraresidual Hα–Hδ, Hα–Hε and Hβ–Hε NOE contacts. Nevertheless, the number of most relevant long range and intersubunit constraints remained virtually equal

The final structural calculations were performed with XPLOR-NIH program to confirm if the NOE distance constraints from automatic procedure were calibrated correctly. The substitution of constraints derived from 3D NOESY with constraints from 4D ^13^C(ali),^13^C(aro)-edited NOESY did not lead to any constraint violations during calculations, or noticeable differences between obtained structures. We thus conclude that the obtained 4D spectrum provides genuine structural information.

## Conclusions

To summarize, we have introduced selective aliphatic–aromatic C,C-edited 4D NOESY experiments. As these experiments inherently lack the diagonal signals, significant spectral artifacts originating from either non-uniform sampling, hardware instability or r.f. pulse imperfections are largely avoided. We have demonstrated that the selective version of aliphatic–aromatic NOESY does not noticeably compromise sensitivity. Thus, the applications of proposed experiment is not limited to small-sized proteins. Owing to high spectral resolution and selection of a subset of NOE contacts it may become a valuable tool for three-dimensional structure elucidation of large proteins. Full benefits of the novel method presented here can be achieved when applied in conjunction with previously reported 4D amide–amide, amide–aliphatic and methyl–methyl NOESY experiments.

## Electronic supplementary material

Below is the link to the electronic supplementary material.

Supplementary material 1 (PDF 1426 kb)
