# Supplementary material for: Selective diagonal-free 13C,13C-edited aliphatic–aromatic NOESY experiment with non-uniform sampling
Source: J Biomol NMR. 2013 May 9;56(3):217–26. doi: 10.1007/s10858-013-9739-5 (PMC3699708; doi:10.1007/s10858-013-9739-5)
Supplement: Supplementary file 1 — Supplementary material 1 (PDF 1426 kb) [file 10858_2013_9739_MOESM1_ESM.pdf]

Selective diagonal-free ^{13}C,^{13}C-edited aliphatic-aromatic NOESY experiment with non-uniform sampling

Jan Stanek[1], Michał Nowakowski[1], Saurabh Saxena[1], Katarzyna Ruszczyńska-Bartnik[2], Andrzej Ejchart[2], Wiktor Koźmiński[1,*]

[1] Faculty of Chemistry, University of Warsaw, Pasteura 1, 02093, Warsaw, Poland

[2]Institute of Biochemistry and Biophysics, Polish Academy of Science, 02106, Warsaw, Poland

Supporting Information

* to whom the correspondence should be addressed

kozmin@chem.uw.edu.pl

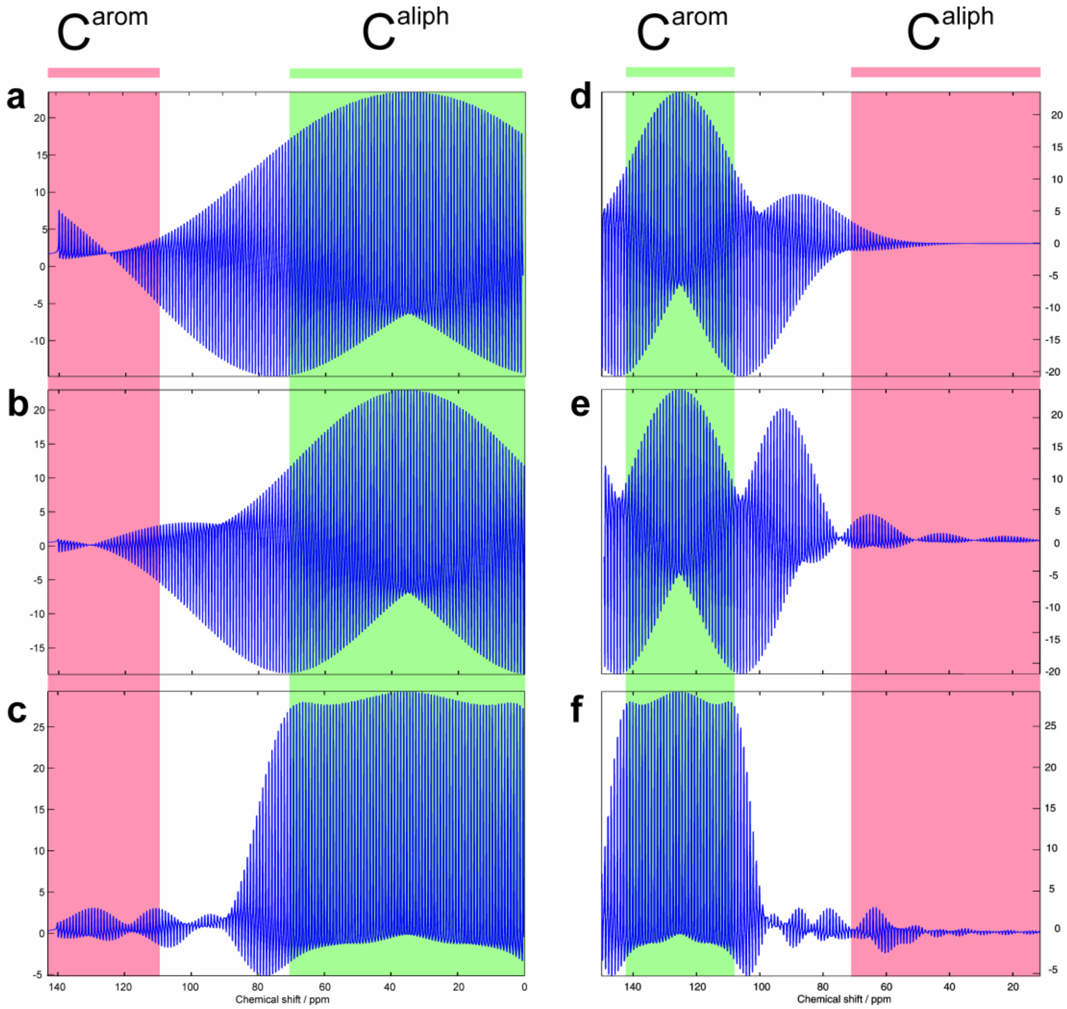

Fig. S1 Shaped ^{13}C $\pi/2$ pulse profiles simulated using Spinach software (Hogben et al. 2011). "Shaped pulse – acquire" sequence was applied to the train of isolated ^{13}C spins of chemical shift ranging from 0 to 150 ppm. Trial pulses for aliphatic-to-aromatic version of ^{13}C,^{13}C-edited NOESY are shown in the left panels (a,b,c). Desired excitation and suppression regions are highlighted in green and pink, respectively. The right panels represent excitation profiles for possible pulses for aromatic-to-aliphatic ^{13}C,^{13}C-edited NOESY (d,e,f). Pulses were applied on-resonance with the carrier placed on 35 and 125 ppm for pulses shown in (a-c) and (d-f), respectively. The following pulse profiles were employed: (a) rectangular pulse of duration of 61μs and peak rf. 4.1 kHz (b) *sinc* (no sidelobes) pulse (95μs, peak rf. 4.1 kHz) (Hutchison et al. 1978); (c) Q5 pulse (Emsley and Bodenhausen 1992) of duration of 393μs and peak r.f. 12.3 kHz; (d) Gaussian pulse of 178μs and peak. r.f 3.4 kHz (e) *sinc* (2 sidelobes) of duration of 276μs and peak rf. 3.71 kHz ; (f) Q5 pulse (786 μs, 6.2 kHz). Effective r.f. field (γB_1) and pulse durations are adjusted to experimental conditions of the sample of S100A1 at B_0=16.4 T (pw90=14.3 μs). Note that despite superior features of Q5 pulses (c,f) their practical performance is worse than corresponding rectangular (a) or Gaussian (d) pulses. The most likely reason for this discrepancy is that neither relaxation nor heteronuclear J_{CH} and homonuclear J_{CC} couplings were included in the simulations and design of shaped pulses.

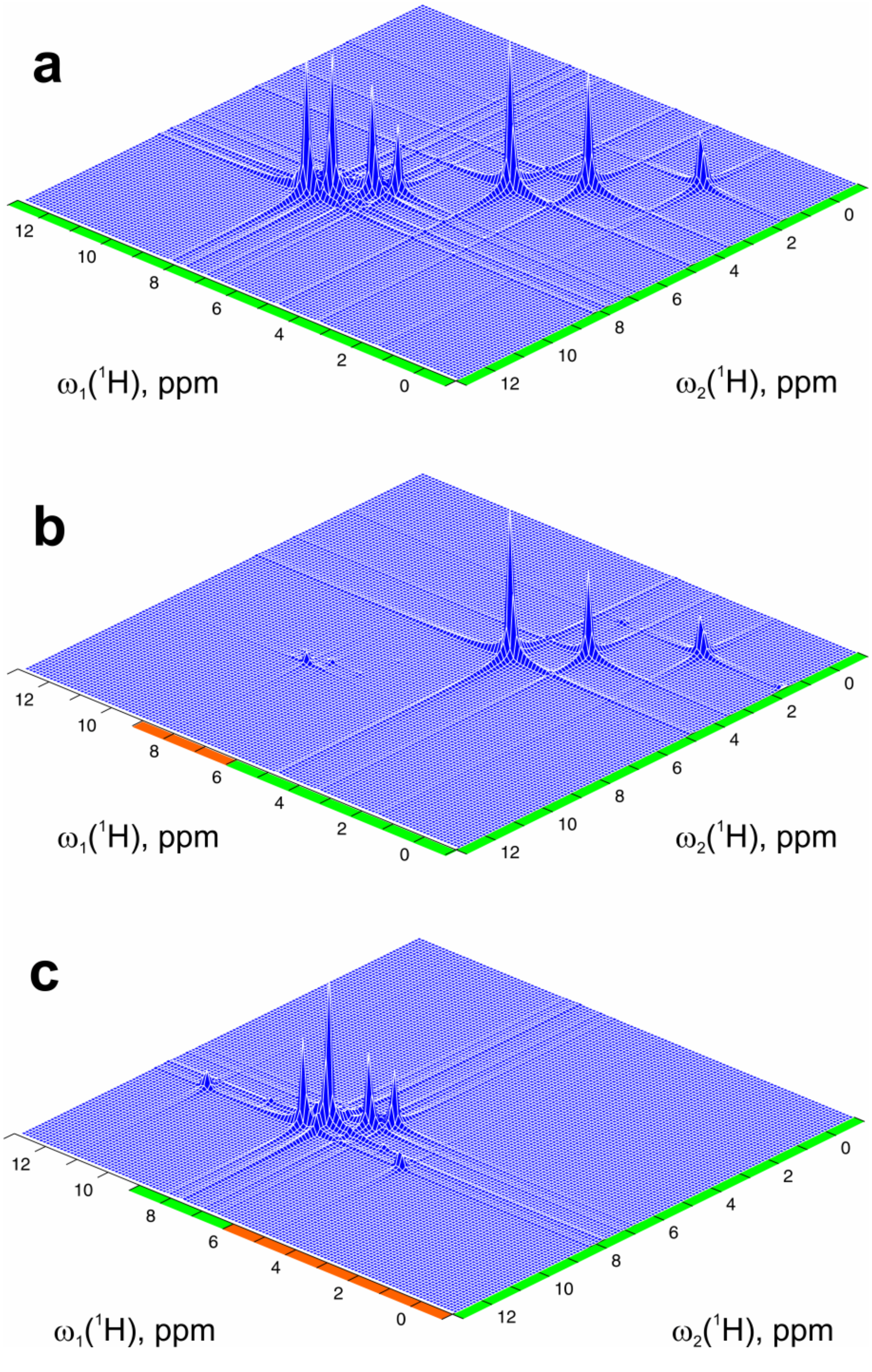

Fig. S2 Simulated 2D ^{1}H-^{1}H correlation spectra obtained using initial HMQC part (including ω_1 (^{1}H) evolution only) of the pulse sequence shown in Fig. 1 followed by z-filtration (PFG), 90°(^{1}H) pulse and acquisition period with idealized ^{13}C decoupling (ω_2 (^{1}H) evolution). (a) Perfect (infinitely broadband) 90°(^{13}C) pulses were applied to obtain uniform excitation. (b) Rectangular pulses with adjusted γB_1 (referred to as ‘A’ and ‘C’ in the pulse sequence

scheme in Fig. 1) applied at the carbon-13 offset of 35 ppm were employed to excite aliphatic and suppress aromatic resonances. (c) Gaussian pulses (referred to as ‘F’ and ‘H’ in the Fig. 1) were applied at the carbon-13 offset of 125 ppm to excite aromatic and discard aliphatic resonances. The simulations were performed using Spinach software (Hogben et al. 2011).

Processing of 4D NUS aliphatic-aromatic NOESY using SSA software

(a) Using native Agilent/Varian input data

The following command

```
cleaner4d --format varian --roi 5.5:9.0 --maxiter 0 ./grid_4400.txt
```

is used for data import, transformation in the direct dimension and extraction of aromatic ^{1}H region (5.5-9.0 ppm), without artefact suppression (no SSA iterations), assuming that the NUS schedule is stored in the file ‘grid_4400.txt’. Subsequently, the reconstruction of spectrum in the Sparky NMR format is accomplished using command

```
reconstructor4d --full --transpose --limits -0.1:5.6,10:70,110:140 roi_5.5_9-nuft
```

where --transpose option and limits for the indirect ^{1}H(aliph), ^{13}C(aliph) and ^{13}C(arom) dimensions are specified to enhance retrieval of spectral regions and minimize size of output file. In the above example it is assumed that the 4D spectrum is analysed using synchronization of aromatic ^{1}H and ^{13}C axes, e.g. with aromatic ^{13}C-HSQC spectrum.

The number of threads used during reconstruction can be manipulated with the environmental variable OMP_NUM_THREADS.

(b) Using input data in the nmrPipe format

It is assumed that input data are stored in the pseudo-2D array in a file, which location is specified in the parameters.txt file. The above discussion remains valid and the only modification necessary is replacement of a --format varian by a --format piperaw option.

For the instructions on preparation of parameters.txt file the reader is referred to the manual of MFT and SSA packages.

References:

Emsley L, Bodenhausen G (1992) Optimization of Shaped Selective Pulses for NMR Using a Quaternion Description of Their Overall Propagators. J Magn Reson 97:135-148

Hogben HJ, Krzystyniak M, Charnock GTP, Hore PJ, Kuprov I (2011) Spinach - A software library for simulation of spin dynamics in large spin systems. J Magn Reson 208:179-194

Hutchison JMS, Sutherland RJ, Mallard JR (1978) NMR Imaging - Image Recovery under Magnetic-Fields with Large Nonuniformities. J Phys E Sci Instrum 11:217-221
